# Pathophysiology and treatment of osteoporosis: challenges for clinical practice in older people

**DOI:** 10.1007/s40520-021-01817-y

**Published:** 2021-03-20

**Authors:** J. Barnsley, G. Buckland, P. E. Chan, A. Ong, A. S. Ramos, M. Baxter, F. Laskou, E. M. Dennison, C. Cooper, Harnish P. Patel

**Affiliations:** 1 Medicine for Older People, University Hospital Southampton NHS Foundation Trust, Southampton, UK; 2 MRC Lifecourse Epidemiology Centre, University of Southampton, University Hospital Southampton NHS Foundation Trust, Southampton, UK; 3 University of Oxford, Oxford, UK; 4 Academic Geriatric Medicine, University of Southampton and University Hospital Southampton NHS Foundation Trust, Southampton, UK; 5 NIHR Biomedical Research Centre, University Hospital Southampton NHS Foundation Trust and The University of Southampton, Southampton, UK

**Keywords:** Bone development, Osteoporosis, Osteosarcopenia, Pathophysiology, Treatment, Older People

## Abstract

Osteoporosis, a common chronic metabolic bone disease is associated with considerable morbidity and mortality. As the prevalence of osteoporosis increases with age, a paralleled elevation in the rate of incident fragility fractures will be observed. This narrative review explores the origins of bone and considers physiological mechanisms involved in bone homeostasis relevant to management and treatment. Secondary causes of osteoporosis, as well as osteosarcopenia are discussed followed by an overview of the commonly used pharmacological treatments for osteoporosis in older people.

**Supplementary Information:**

The online version contains supplementary material available at 10.1007/s40520-021-01817-y.

## Introduction

Osteoporosis, a disease characterised by low bone mass and microarchitectural deterioration of bone tissue is the most common chronic metabolic bone disease. Annually contributing to 8.9 million fractures worldwide as well as to reduced physical and psychological health, lower quality of life and shorter life expectancy, osteoporosis represents a major global health problem [1–3]. In the UK, over 300,000 patients present to hospitals with fractures associated with osteoporosis and this is associated with a high health care cost [4]. For example, In the year 2000, osteoporosis incurred an estimated £1.8 billion in UK health costs and is predicted to increase to £2.2 billion by 2025. The prevalence of osteoporosis increases with age and both older women and men are at higher risk of fractures associated with both osteopenia and osteoporosis. These commonly occur at the vertebrae, wrist, hip and pelvis following low energy transfer trauma such as falling from a standing height—termed fragility fractures. Octo- and nonagenarians bear the greatest burden of osteoporosis related fractures and consequent morbidity and mortality. For example, mortality rate can be up to 20% in the years following hip fracture. Specific morbidity includes disability, chronic pain, impaired function and loss of independence and risk of short- and longer-term institutionalisation.

To understand the mechanism by which osteoporosis develops and the treatment options available, an understanding of the development, structure and remodelling process of bone in addition to the effects of ageing, disease and drug treatments on bone is needed. In this narrative review, our aim is to explore bone physiology and homeostasis, pathology and diagnosis of primary and secondary osteoporosis, osteosarcopenia and management of osteoporosis relevant to clinical practice.

## Origins of bone, structure, function and differences in biological sex

During gastrulation, the blastula differentiates into three distinct cell lineages: the ectoderm, mesoderm and endoderm. At week four, the mesenchymal cells of the intraembryonic mesoderm divide into the three paired regions: paraxial mesoderm, intermediate mesoderm, and the lateral plate mesoderm [5]. The lateral plate mesoderm eventually forms the limb skeleton. The paraxial mesoderm segments into somites, which subdivide into sclerotomes, myotomes, syndetomes and dermatomes. The paraxial mesoderm hence gives rise to the muscles and axial skeleton [5]. The development of bone and muscle are intrinsically linked given their common embryonic origins (supplementary Fig. 1).

**Fig. 1:**
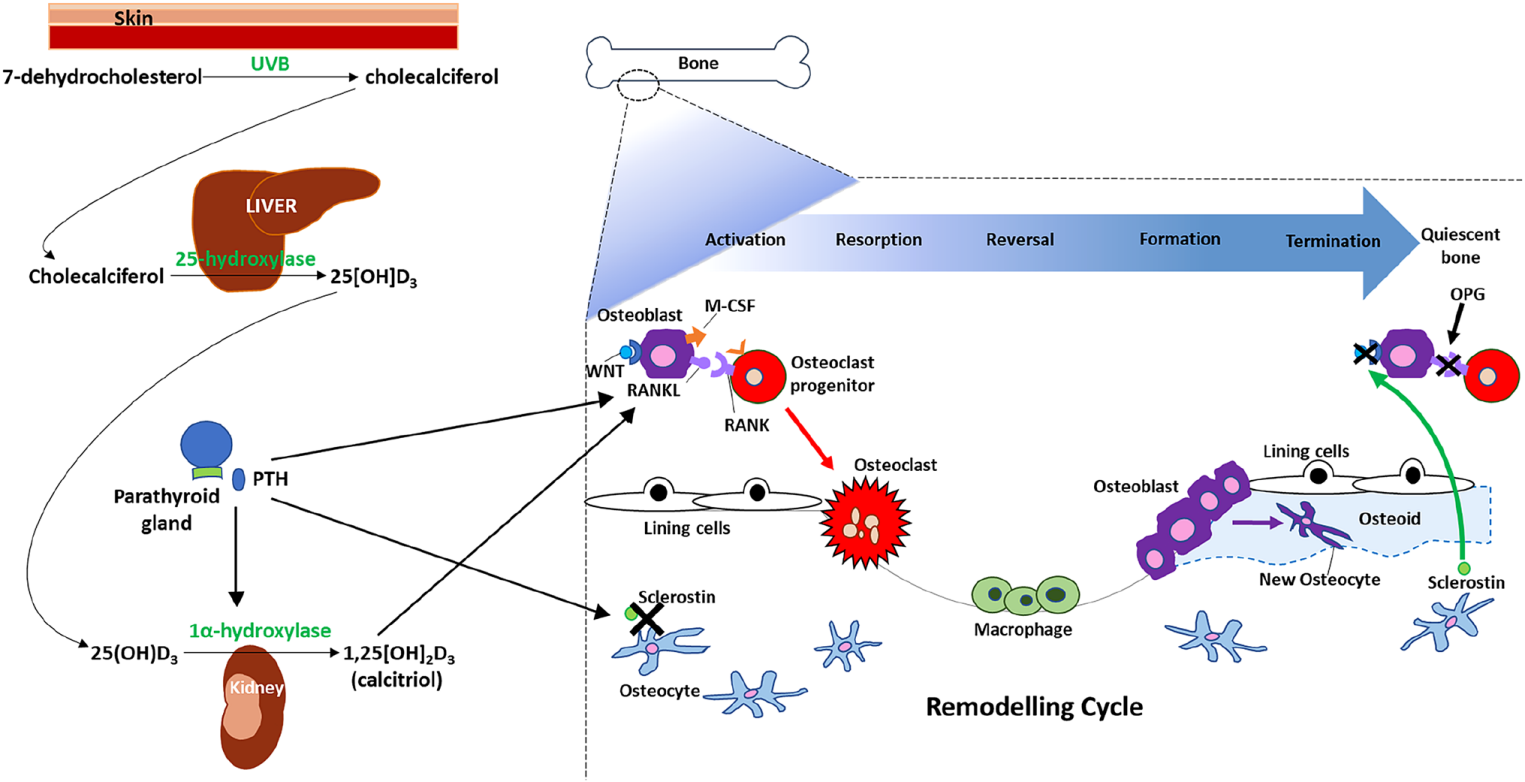
Remodelling cycle and regulators of bone formation. The bone remodelling cycle occurs in 5 stages—activation (during which osteoblastic expression of M-CSF and RANKL stimulate osteoclast progenitor maturation and differentiation into osteoclasts), resorption of bone (by osteoclasts), reversal, formation (new bone laid down by osteoblasts) and termination (bone returns to quiescent phase). Bone remodelling is stimulated by calcitriol and PTH and is inhibited during the quiescent phase by sclerostin, which inhibits WNT driven bone formation and OPG which inhibits RANK-RANKL interactions

### Bone structure

Bone is a specialised and multifunctional connective tissue with both an organic and inorganic component. The organic bone matrix (osteoid) is comprised of collagenous proteins, the predominant being type I collagen [6] as well as a broad range of non-collagenous proteins including glycosaminoglycans, glycoproteins and some serum derived proteins. The numerous non-collagenous proteins regulate aspects of bone metabolism including deposition, mineralisation and turnover [7]. The inorganic component predominantly consists of calcium and phosphorus in the form of hydroxyapatite and provides mechanical rigidity to the bone and contributes to 50–70% of the overall total bone mass [6].

Of the two main types of bone in the adult skeleton, cortical comprises approximately 80% of adult bone mass and trabecular, the remaining 20%. Cortical bone is dense, has a low turnover rate of around 3% per year and maintains mechanical strength and integrity of the bone [6, 8]. In contrast trabecular bone, found in long bones and vertebrae has a turnover rate of approximately 26% per year, has a lower mineralised content, is more metabolically active and responsive to hormonal stimuli [8]. Trabecular bone undergoes remodelling more than cortical bone; the clinical relevance is that fragility fractures typically occur in trabecular bone [8].

The cellular component of bone is chiefly composed of three cells: osteocytes, osteoblasts, and osteoclasts. Osteocytes are found in the lacunae of the matrix and have a mechano-sensory function in bone formation. Osteoblasts synthesize osteoid whilst osteoclasts enzymatically resorb bone [5]. All three subtypes are important for bone growth and remodelling throughout the lifecourse.

### Bone homeostasis

Bone remodelling allows repair of micro-damage, maintaining skeletal structure as well as serum calcium and phosphate homeostasis and involves a careful equilibrium between the action of osteoclasts, tightly coupled with that of osteoblasts. Numerous clusters of these cells within multicellular units are found along the bone surface forming active remodelling sites which are individually covered by a cell canopy. This cell canopy has been found to be derived from mesenchymal stem cells (MSC) surrounding the red bone marrow and acts as a source of progenitor cells during the remodelling process [9]. A remodelling cycle on the resting bone surface occurs through five sequential stages: activation, resorption, reversal, formation and termination [10] (Fig. 1).

### Activation

Activation of the resting bone surface is mediated by osteocytes that express an amino acid peptide; receptor activator of nuclear factor (NF) Kappa-B ligand (known commonly as RANKL), which interacts with the RANK receptor on osteoclast precursors that potently induces differentiation into multinucleated osteoclasts. Osteoblast expression of macrophage colony-stimulating factor (M-CSF) also promotes osteoclast precursor survival and differentiation. Osteoblasts produce chemokines, to recruit osteoclast precursors, and matrix metalloproteinases to degrade un-mineralised osteoid and expose adhesion sites for osteoclast attachment [11–13].

### Resorption

Osteoclasts secrete hydrogen ions and lysosomal enzymes, e.g., cathepsin-K into a ‘sealed zone’ beneath the cell [14, 15]. Through acidification and proteolysis, they remove a tunnel of old bone. Osteoprotegerin (OPG) can block the RANK-RANKL interaction, thus reducing resorption by inhibiting osteoclast differentiation and increasing their apoptosis [11].

### Reversal

The reversal phase has been subject to intense research in recent years [16, 17] and begins with osteoclastic signalling that persists for approximately 4 to 5 weeks [18] and is ultimately responsible for the crucial coupling of osteoclastic and osteoblastic activity seen at remodelling sites. ‘Reversal cells’ have long been recognised and although clearly distinct from osteoclasts and osteoblasts, their exact morphology and function is still uncertain [19].

### Formation

Osteoblasts deposit un-mineralised osteoid until the tunnel of resorbed bone is completely replaced, resulting in minimal net change in bone volume during remodelling [20]. Bone formation is complete as osteoid is gradually mineralised through incorporation of hydroxyapatite. By the end of bone formation, approximately 10 to 15% of mature osteoblasts are entombed by the new bone matrix and differentiate into osteocytes. At rest osteocytes express sclerostin, which prevents WNT signalling (an inducer of bone formation) in osteoblasts [21]. Sclerostin expression is inhibited by parathyroid hormone (PTH) or mechanical stress, allowing wnt-induced bone formation to occur [12].

### Termination

When the tunnel of resorbed bone has been fully replaced, the remodelling cycle ends through a series of yet undetermined termination signals. The resting bone surface is re-established.

### Regulation of bone remodelling

Remodelling signals may be hormonal or mechanical in nature. Systemic regulators of bone formation include oestrogen, growth hormone and androgens. Thyroid hormones are essential for normal musculoskeletal development, maturation, metabolism, structure and strength where they promote bone turnover by influencing osteoblast and osteoclast activity [22]. Glucocorticoids prolong osteoclast survival and reduce bone formation by increasing osteoblast apoptosis [23]. Continuous high-dose parathyroid hormone (PTH) release induces bone resorption indirectly by promoting RANKL/MCSF expression and inhibiting OPG expression (14). Meanwhile, low intermittent PTH release induces bone formation by promoting increased survival, proliferation and differentiation of osteoblasts [24]. Other systemic regulators of bone remodelling include vitamin D3, calcitonin, insulin-like growth factor, prostaglandins and bone morphogenetic proteins. Local regulators of bone remodelling include cytokines, growth factors such as IGF-1, Sirtuins, protein kinases such as mechanistic target of rapamycin (mTOR), Forkhead proteins, M-CSF, wnt, sclerostin, and the RANK/RANKL/OPG system [12, 24]. Bone remodelling is tightly controlled and alterations in cellular activity, i.e., increased osteoclastic activity in response to extrinsic or intrinsic cues will lead to increased bone resorption and decreased bone formation.

Bone volume and mass decline in older individuals and in all ethnicities. An imbalance in remodelling within the aged microenvironment is driven by MSC senescence and a shift in differentiation potential to favour adipogenesis within the bone marrow. An altered intracellular signalling milieu such as lower Sirtuin levels can lead to an increase in sclerostin activity, with inhibition of wnt and suppression of bone formation. Increased activity of mTOR translates to increased osteoclastic activity and release of cathepsin K [25].

### Calcium and vitamin D homeostasis

Sufficient calcium supply is essential for bone mineralisation. Bone also acts as a calcium reservoir, restoring physiological homeostasis when serum levels are low through the action of PTH on bone resorption, renal calcium reabsorption and synthesis of active vitamin D [26]. Inactive vitamin D is hydroxylated first by 25-hydroxylase (CYP2R1) in the liver and is then converted to its active form, calcitriol (1,25[OH]_2_D_3_), in the kidneys by 1a-hydroxylase (CYP27B1). PTH stimulates 1α-hydroxylase to increase levels of calcitriol. When serum calcium levels are normal or low, calcitriol acts on vitamin D receptors (VDRs) to increase intestinal and renal calcium uptake. However, when dietary calcium is insufficient to meet calcium demand, i.e., during periods of undernutrition often seen in older people, a negative calcium balance ensues. At this juncture, calcitriol inhibits bone mineralisation and enhances bone resorption through upregulation of RANKL expression. Through these actions, calcium and phosphate are mobilised from bone matrix to serum, at the expense of skeletal integrity. Calcitriol activation of osteocyte VDRs results in increased production of fibroblast growth factor 23 (FGF-23) which inhibits 1α-hydroxylase, thus creating a negative feedback system [27].

### Peak bone mass and differences in biological sex

Peak bone mass is defined as the maximum amount of skeletal tissue an individual will have in their life at the termination of skeletal maturation. Peak bone mass is thought to be attained between 25 and 30 years of age thereafter decreasing at a rate of 0.5% per year [28]. Males attain a higher BMD, albeit later than females. The attainment of peak bone mass is a multifactorial process. The strongest evidence for peak bone mass appears to be genetically determined; up to 85% of the variation on peak bone mass can be explained by genetic factors, which in turn affects the physiological metabolism of bone [29]. It has been hypothesised that a rise in IGF-1 during puberty results in increased plasma inorganic phosphate and calcitriol, leading to increased bone mass gain during puberty. Patients with haploinsufficiency of IGF-1 receptor have been found to have undesirable changes to bone architecture in accordance with this hypothesis [30, 31]. Other factors that can influence peak bone mass include nutrition (calcium and vitamin D status), physical activity, inter-current illness and socioeconomic deprivation.

## Osteoporosis

Bone loss is an inevitable consequence of ageing. Conditions which hinder an individual’s ability to maximise peak adult bone mass, increase the probability of developing osteoporosis and elevate fracture risk later in life. Primary osteoporosis can be categorised into age-related or post-menopausal. Women have an increased risk of primary osteoporosis, insofar as they reach a lower peak bone mineral density in comparison to men. This risk is further increased by the post-menopausal decline in oestrogen. However, it is important to note that approximately 20% of men with osteoporosis are hypogonadal.

Bone loss in women is most evident in the trabecular vertebral bodies as they are more metabolically active and are sensitive to the trophic effects of oestrogen which has a significant role in preventing bone resorption by inhibiting osteoclasts [32]. A steeper decline in bone mass begins approximately between 65 and 69 years in women and between 74 and 79 years in men [28]. Women aged 50 or over have a four-fold higher rate of osteoporosis and two-fold higher rate of osteopenia than men [2]. The lifetime risk of osteoporotic fractures in women is approximately 40% [33]. Weight loss in older people, smoking and moderate to high alcohol intake appear to accelerate the loss of bone in both men and women.

### Secondary causes of osteoporosis relevant to older people

Several illnesses associated with osteoporosis are listed in Table 1. A few of those illnesses and drug therapies pertinent to the development of osteoporosis in older people are discussed below. Further detailed discussion of secondary osteoporosis can be found in an excellent review [34].

**Table 1:** Secondary causes of osteoporosis relevant to older people

**Glucocorticoid induced osteoporosis**	Monoclonal gammopathy
**Proton pump inhibitor use**	Multiple myeloma
**Antiepileptic drug use**	Hyperparathyroidism
**Systemic hormonal therapy**	Chronic liver disease
**Selective serotonin re-uptake inhibitor use**	Ankylosing spondylitis
**Disorders of the thyroid**	Coeliac disease
**Loop diuretic use**	Rheumatoid arthritis
**Chronic kidney disease**	Multiple sclerosis
**Diabetes**	

^*^Other risk factors for osteoporosis relevant for older people include prolonged immobility, previous fragility fracture, height loss > 3–5 cm, BMI < 19 kg/m^2^, consumption of ≥ 3 units of alcohol/day, current smoking

^*^The most common causes pertinent to older people are in bold and discussed in text

### Glucocorticoids

**Fig. 2:**
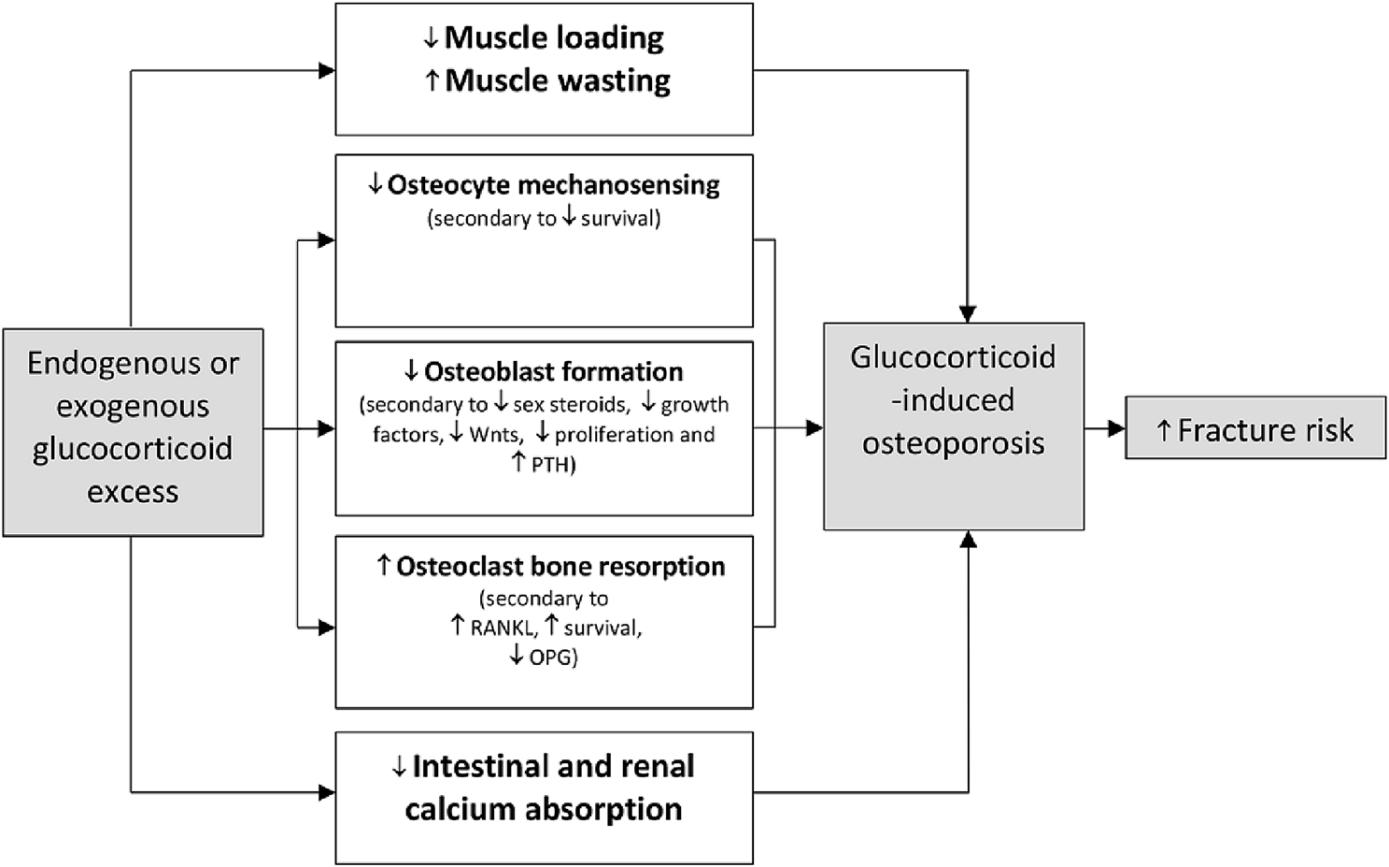
Actions of glucocorticoid excess on bone

Glucocorticoids form part of the treatment strategy in a wide variety of diseases, including chronic inflammatory, rheumatological and respiratory illnesses. It is estimated that 1% of the population in the United Kingdom are receiving long term glucocorticoid therapy and the rate of long-term use is gradually increasing [35]. However, the anti-inflammatory therapeutic benefits are accompanied by adverse effects on bone health. It is estimated that vertebral and non-vertebral fractures occur in 30–40% of patients who are receiving chronic glucocorticoid therapy, and this effect appears to be dose dependent [36]. Nevertheless, fracture risk can rapidly return to baseline after steroid cessation. Glucocorticoids impair the function of osteoblasts, induce apoptosis of both osteoblasts and osteocytes and promote osteoclast formation ultimately leading to net suppression of bone formation [37]. The hormonal and intracellular effects of glucocorticoids are summarised in Fig. 2.

### Proton pump inhibitors (PPI)

Long-term PPI prescriptions in the UK are increasing and their use have been associated with an elevated fracture risk [38]. Two main mechanisms are involved. Firstly, hypergastrinemia leads to osteoclastic precursor stimulation resulting in an alteration in balance that favours increased bone resorption. Secondly, hypochlorhydria affects the absorption of calcium and magnesium, leading to hyperparathyroidism and an increase in osteoclastic activity. There is an overall increase in fracture risk through the effects on bone remodelling, decreased mineral absorption as well as a decrease in muscle strength leading to poorer physical function and a higher falls risk [38].

### Antiepileptic drugs (AED)

A systematic review and meta-analysis of 22 studies demonstrated that the use of AED is associated with an 86% increase in the risk of fractures at any site and a 90% increase in the risk of hip fractures [39]. This risk is higher in users of liver-enzyme inducing AED compared to non-enzyme inducing AED. Examples include phenobarbiturates, topiramate and phenytoin. Several theories are proposed to explain the effect of AED on bone metabolism. Predominantly, AED activate the orphan nuclear and pregnane-X receptors (PXR—expressed in the gut, kidneys and liver) and induce the cytochrome P450 enzyme system (CYP2, CYP3) leading to increased metabolism of vitamin D to inactive metabolites. Deficiency of vitamin D then causes hypocalcaemia and secondary hyperparathyroidism resulting in low bone mineral density and bone loss [40].

### Systemic hormonal therapy

Aromatase inhibitors (AIs) used in the treatment of breast cancer can lead to increased bone loss and negative bone balance due to severe oestrogen depletion [41]. The increased bone loss from AI use compared to physiological postmenopausal bone loss is at least two fold [42]. Gonadotrophin Releasing hormone (GnRH) agonists bind to GnRH receptors in the pituitary and downregulate the gonadotropin-producing cells, limiting luteinizing hormone and follicle stimulating hormone secretion. Administration then leads to lower production of testosterone and oestradiol. Osteoblast, osteoclasts and osteocytes express androgen and oestrogen receptors and are responsive to both sex hormones. Reduction of these hormones leads to cellular dysfunction and affects bone remodelling by promoting bone resorption over formation [43].

### Selective serotonin reuptake inhibitor (SSRI)

Osteoblasts and osteocytes harbour serotonin (5-hydroxytryptamine [5-HT]) receptors. 5-HT may have important signalling and regulatory roles in bone remodelling. SSRI use in women with a mean age of 78.5 years was associated with an increased rate of non-spine fractures [44]. In older men who took SSRI, a decrease of 3.9% in bone mineral density compared to those taking tricyclic antidepressants or none was observed [45]. The exact mechanisms of SSRI associated increase in fracture risk are currently still unclear. However, it is theorized that disruption of the serotonin receptors in bone cells could lead to altered signalling of bone formation pathways favouring bone resorption [46].

### Thyroid disease

Hyperthyroidism can lead to higher bone turnover and osteoporosis. Both endogenous (primary thyroid disease) and exogenous causes, i.e., long term therapeutic use of levothyroxine as well as over-replacement with levothyroxine is associated with lower BMD and a subsequent increased risk of hip and vertebral fractures [22, 34, 47]. Hypothyroidism is associated with a slowing of bone formation and resorption and does not appear to increase fracture risk. However, subclinical hypothyroidism is associated with lower BMD and increased fracture risk in post-menopausal women [22].

### Loop diuretics (LD)

LD are commonly prescribed and used in older people. Their diuretic activity centres around sodium and chloride reabsorption at the loop of Henle but they also decrease calcium reabsorption and increase calcium excretion. Hypocalcaemia can lead to increased bone turnover and lower BMD [48]. In clinical studies, use of LD was associated with lower hip BMD in men and higher fracture risk in post-menopausal women [49]. However, longer term studies and pragmatic RCTs are needed to study these effects as renal calcium loss may be offset by PTH dependent increase in active vitamin D activity to maintain normocalcaemia [50].

### Chronic kidney disease (CKD)

The incidence of CKD increases in older age, but also in those who are hypertensive and have diabetes. CKD is associated with osteoporosis and renal osteodystrophy through perturbations in 1α-hydroxylation of vitamin D in the kidney, hypocalcaemia and hyperparathyroidism. Detailed discussion on renal bone disease is out of scope for this review. Several recent articles offer detailed reviews [51].

### Diabetes

Both type 1 (T1DM) and type 2 (T2DM) diabetes are risk factors for low-energy fractures as both types are associated with low bone quality and strength, though only T1DM typically reduces BMD. T1DM is associated with increased risk of fracture throughout the lifecourse particularly at the hip [52], even when accounting for co-morbidities such as chronic kidney disease [53]. Fracture risk is hypothesised to be in part secondary to a deficiency in insulin and IGF-1, which are known to be important in determining peak bone mass [54]. T1DM commonly develops in adolescence and early adulthood. This represents a critical time to implement strategies to improve BMD and maximise peak bone mass.

T2DM is not associated with decreased BMD but is still associated with increased fracture risk at the hip, vertebrae and other vulnerable sites [52]. This presents a clinical challenge in older people insofar as the gold standard measurement of BMD may underestimate the true fracture risk for patients with T2DM. In fact, increased mechanical loading most often secondary to obesity and hormonal factors such as hyperinsulinemia, favour increasing deposition of bone [55]. As such, other mechanisms contribute to the increased fracture risk. For example, increased production of advanced glycation end-products in patients with chronic hyperglycaemia impair collagen cross-linking in the bone matrix, reducing bone quality and strength [56]. Patients with diabetes are also at higher risk of falling, attributable to factors such as peripheral neuropathy, lower physical function, orthostatic hypotension, poorer eyesight as well as hypoglycaemia from pharmacotherapy.

### The coexistence of osteoporosis and sarcopenia—osteosarcopenia

Given bone and muscle share similar embryonic origins, both tissues may be influenced by similar metabolic (endocrine and paracrine) and environmental cues to maintain homeostasis. Sarcopenia (muscle failure) is characterised by a decline in skeletal muscle strength, mass and function [57]. Primary sarcopenia occurs with advancing age, whilst secondary sarcopenia is secondary to co-existent illnesses, e.g., diabetes. The prevalence of sarcopenia increases with age and similar to osteoporosis has a multifactorial aetiology—undernutrition, decreased physical activity, inflammation, presence of comorbid diseases. Diagnosis of sarcopenia involves measuring muscle strength (hand grip strength) and function (walking speed or chair rise time) [58, 59], as well as lean mass—Dual-energy X-ray Absorptiometry (DXA) in this situation is a useful method to assess both total and appendicular lean mass as well as a bone mineral density for those suspected to have osteosarcopenia.

The pathophysiology of osteosarcopenia is multifactorial; the common mesenchymal origins of bone and muscle infer a close relationship in their pathogenesis [60]. As such, similar genetic factors can have a pleiotropic influence on both bone and muscle. Polymorphisms of several genes including androgen receptor, oestrogen receptor, IGF-1, and vitamin D receptor have been identified that can influence molecular cross talk and alter cellular mechanisms resulting in an imbalance in muscle and bone turnover [61]. The biomechanical relationship of muscle and bone is evident during ageing where lower physical activity and mechanical loading contributes to both decreased muscle mass, function and bone mineral density. This supports the ‘mechanostat hypothesis’, which postulates that if the mechanical forces of the skeletal musculature acting upon the periosteum reach a given threshold, growth is stimulated as opposed to bone resorbed [62]. In addition, as with caloric intake, dietary vitamin D and protein intake also diminishes with age, contributing to reduced muscle strength, lower bone mineralisation and an increase in falls risk [63]. Osteosarcopenia represents an additive burden for older people in terms of their physical and psychological health as well as their quality of life. Understanding the pathophysiology of osteosarcopenia is key to informing combined strategies for treatment and prevention.

## Osteoporosis: diagnosis and management

The diagnosis of osteoporosis is made using DXA scanning to measure the bone mineral density (BMD) of the proximal femur to obtain a T-score. The T-score represents the number of standard deviations (SD) a patient’s BMD is below the mean reference value of a healthy young population. A T-score ≤ 2.5 SD below the reference value indicates osteoporosis [61] and where this is accompanied by one or more fractures, this indicates severe osteoporosis. However, the majority of fractures occur in individuals who are osteopenic, defined by a T-score of between 1.0 and 2.5 SDs below the mean reference value. These criteria, when combined with ascertainment of other risk factors and patient preferences inform appropriate lifestyle-management and treatment strategies. Interpretation of DXA results older people should be interpreted in context of the coexistence of degenerative spine disease, vertebral collapse, disc disease that can artificially elevate BMD. Conversely in osteomalacia, a complication of malnutrition in older people, lower total bone matrix and can lead to underestimation of BMD [64].

### Assessment of risk

The UK National Institute for Health and Care Excellence (NICE) advise that all women aged 65 and above, all men aged 75 and above, and younger patients with risk factors should receive a form of osteoporosis risk assessment. The gold standard investigation for osteoporosis is bone mineral density (BMD). BMD at the femoral neck, age, sex, smoking, family history, and the use of oral glucocorticoids can be used to calculate the FRAX score. This tool estimates the 10-year probability of osteoporotic-related fracture [65, 66]. The Trabecular Bone Score and QFracture are other assessment tools which have shown good predictive value [67]. All risk calculators generate a probability risk rather than indication for treatment. Whether in the community or within secondary care, management should be patient centred with treatment decisions based on shared decision making and what matters most for the patient with respect to patient preference, presence of comorbid diseases, i.e., CKD, consequent polypharmacy burden, social and psychological circumstances. It is worth noting that the FRAX score does not incorporate the dose-dependent effect of corticosteroids, alcohol and smoking on fracture risk nor the increased risk incurred by multiple prior fractures. These factors should be taken into account when assessing an older person’s individual fracture risk [64].

Screening and intervention for individuals who are high risk of fracture as a primary preventative endeavour could reduce the burden of future fragility fracture. For example, screening with FRAX and pharmacological intervention for post-menopausal women aged 70–85 at high risk developing a fracture was associated with a decrease in hip fracture rate and was deemed to be cost effective compared with usual care in the UK SCOOP study [68]. Older people presenting to secondary care with hip fracture are likely to be osteoporotic, sarcopenic, and also have several markers of frailty. As such their assessment and management should be multidisciplinary (orthopaedic, older people’s specialist teams, pharmacy, therapy, nursing, mental health, dietetics, speech and language) and be driven by the process of comprehensive geriatric assessment (CGA) [69]. Whilst this is the gold standard for patients presenting with a hip fracture, for less frail and more ambulant individuals presenting with other fragility fractures, i.e., wrist, shoulder and vertebral, fracture liaison services (FLS), which are typically multidisciplinary co-ordinated models of care systematically assess, identify and advise on risk factor management. They have a vital role in reducing the risk of subsequent, more debilitating fractures. Global Initiatives such as the International Osteoporosis Foundation’s Capture the Fracture initiative (capturethefracture.org) support the expansion of FLS within secondary care institutions. General principles include preserving bone mineral density, preserving muscle strength, and managing falls and other risk factors to maintain an individual’s independence.

Recently the concept of imminent fracture has been developed to highlight those most at risk of fracture within 2 years after a sentinel fracture. These events can occur in up to 23.2% of patients [70] and risk factors include recent fracture, fracture site, older age, osteoporosis and comorbidities, e.g., cognitive dysfunction, central nervous system polypharmacy, reduced physical activity, poorer general health as well as falls [71, 72]. This supports the notion of early identification, assessment and treatment of those most at risk with the FLS model to reduce the future burden of fracture [73].

The diagnosis of sarcopenia relies on case finding through administering the SARC-F questionnaire or determining the presence of weaker grip strength through hand-held dynamometry or poorer performance in repeated chair rises. A probable case of sarcopenia identified at this stage allows multicomponent intervention with either nutritional or physical activity interventions. Measurement of lean mass through DXA and other measures of physical function such as gait speed can determine whether an individual has severe sarcopenia [59].

### Non-pharmacological options for the treatment of osteoporosis

Research supports physical activity and exercise for the prevention of osteoporosis and related injury from falls and fractures [74, 75]. In addition to preserving skeletal muscle, resistance exercise has also been shown to increase bone strength through repeated mechanical loading, thereby improving bone mineral density and reducing the development of osteoporosis [76]. For example, a systematic review of 43 randomised controlled trials and found the most effective type of exercise for increasing neck of femur bone mineral density was high force exercise, such as progressive resistance strength training of the lower limbs [77]. In addition, correcting biomechanical imbalance in the abdominal trunk as well as strengthening hip flexion and knee extension has been shown to reduce the risk of falls and alleviate musculoskeletal pain [78–80]. Furthermore, smoking cessation, avoiding alcohol excess, optimising dietary intake of calcium and consuming a balanced diet rich in fruit and vegetables, with a slant towards an increased protein intake are modifiable factors contributing to the prevention of osteoporosis [81–83]. In general, these principles can also apply to the management of sarcopenia and by reducing the risk of falls and subsequent fracture through improved bone mineral density, acute decompensation and progression of the frailty syndrome can be mitigated [84, 85].

### Pharmacological options for the treatment of osteoporosis

There are various pharmacological options for osteoporosis treatment that aim to reduce the risk of fractures. These include:

- 1. Calcium and vitamin D
- 2. Antiresorptive therapy—Bisphosphonates, Denosumab.
- 3. Hormonal treatment—Selective oestrogen receptor modulators, Testosterone, PTH analogues.
- 4. Novel therapies—Romosozumab, Dickkopf-1 (Dkk1) inhibitors.

#### 1. Calcium and Vitamin D

Vitamin D deficiency in older people is common, not only secondary to physiological changes in the ability of the skin to synthesise vitamin D but particularly in those who are malnourished, have chronic kidney disease, are institutionalised or are housebound. National guidance recommends 1000 mg of calcium in combination with 400 International Units (IU) of vitamin D per day. Housebound older people or those living in a nursing home are advised to take 800 IU of vitamin D per day. A meta-analysis found that calcium and vitamin D supplementation reduced the risk of hip fracture by 30% and the total fracture risk by 15% [86]. This was supported by a study which found a 12% reduction in all fractures and a reduced rate of loss of BMD in the hip and spine in patients taking a minimum dose of 1200 mg calcium and 800 IU of vitamin D [87] (Table 2).

**Table 2:** Advantages, adverse effects and contraindications of the main pharmacological interventions in osteoporosis

Treatment	Advantages	Side effects and contraindications	Guidance	Posology
Calcium and vitamin D	Reduce the risk of hip fracture and of total fracture Reduce rate of loss of BMD in the hip and spine Reduction in risk of falling Favourable effects on muscle health	Gastrointestinal symptoms Renal stones Some evidence suggests an increased risk of cardiovascular disease (including myocardial infarction) *Contraindicated* in pre-existing hypercalcaemia	Housebound older people or those living in a nursing home are advised to take 800 IU of vitamin D per day	1000 mg of calcium in combination with 400 IU of vitamin D, daily
Bisphosphonates	Reduce the risk of fractures amongst a broad age range of patients Increase BMD (lumbar spine; trochanter; femoral neck; total proximal femur) Reduce the risk of mortality when commenced after a fracture	Gastrointestinal symptoms Bone/muscle/joint pain Hypocalcaemia Osteonecrosis of the jaw (rare) Atypical femoral fractures (after 5 years of use) Issues with adherence *Contraindicated* in severe renal impairment; conditions impairing gastric emptying (achalasia, oesophageal stricture) and hypocalcaemia	For postmenopausal women and men over 50 years of age, who have been confirmed by DXA scan to have osteoporosis Ensure patients have normal serum calcium levels and are replete in vitamin D. Advise good dental hygiene and well-fitting dentures	Alendronate 10 mg PO, once daily or 70 mg once weekly Risedronate 5 mg PO, once daily or 35 mg PO, once weekly Ibandronic acid 150 mg PO, monthly (3 mg every 3 months IV) Zoledronic acid 5 mg IV annually
Denosumab	Reduce the risk of fractures (vertebral, hip, non-vertebral fractures) Increase in BMD without plateau	Hypocalcaemia (especially if impaired renal function) Increased risk of bacterial infections Skin rash Increase fracture risk back to pre-treatment level Osteonecrosis of the jaw (rare) Atypical femoral fractures Risk of severe hypocalcaemia especially with coexistent Vitamin D deficiency as well as renal impairment	Alongside calcium and vitamin D supplementation Alternative when oral bisphosphonates are not tolerated or are contraindicated Ensure patients have normal serum calcium levels and are replete in vitamin D. Advise good dental hygiene and well-fitting dentures	60 mg SC (6-montly)
Selective oestrogen receptor modulators	Reduction in both vertebral and non-vertebral fracture risk Appear to be safe to use in older people	Hot flushes; Lower limb cramps; Joint pain; Can increase risk of venous thromboembolism	Treatment and prevention of osteoporosis in post-menopausal women and are indicated after first line therapies have been considered	Raloxifene 60 mg, daily
Testosterone	Increase in BMD in men who are hypogonadal	Aggression; Prostate cancer; Psychiatric symptoms	For men at high risk for fracture with testosterone levels below 200 ng/dl (6.9 nmol/litre) and have contraindications to other osteoporosis’ therapies	
Teriparatide	Appears to increase bone formation within 24 months of use	Nausea; Pain in limbs; Headache; Dizziness; *Contraindicated* in conditions with increased bone turnover (pre-existing hypercalcaemia, hyperparathyroidism, Paget’s disease); unexplained raised alkaline phosphatase and previous bone radiation therapy; malignancies with bony metastases and severe renal impairment	If intolerant or suffer severe side effects from first line therapies described	20 mcg SC daily (max. 24 months)
Abaloparatide	Lower risks of new vertebral fractures (compared teriparatide) Reduction in risk of nonvertebral Increase in BMD	Appears safe. Further drug safety data pending	Headache, nausea, dizziness, joint pain are key adverse effects	80 mcg SC daily
Romosozumab	Reduces the risk of vertebral and nonvertebral fractures	ARCH study reported an imbalance in serious cardiovascular adverse events. Use may be restricted	Joint pain, hypersensitivity reactions, hypocalcaemia are key adverse effects	210 mg (2 × 105 mg injections) SC monthly for 12 months

*BMD* bone mineral density, *IU* International Units, *mg* milligrams, *DXA* dual-energy X-ray absorptiometry, *PO*: per oral, *IV*: intravenous, *SC* subcutaneous

Evidence opposing the use of calcium supplementation suggests an increased risk of cardiovascular disease, including myocardial infarction [88]. However, other studies found no association between calcium supplementation and risk of cardiovascular disease [89, 90]. Overall, there is insufficient evidence to outweigh the benefits from supplementation and current guidance recommends supplementation should be given to those with increased risk of insufficiency and individuals receiving treatment for osteoporosis. Calcium and vitamin D supplementation have also shown to have favourable effects on muscle health and the reduction in risk of falling [91].

#### 2. Antiresorptive therapy—Bisphosphonates (Alendronate, risedronate, ibandronate and zolendronic acid)

Bisphosphonates bind strongly to hydroxyapatite, inhibit osteoclast-mediated bone resorption and increase bone mineral density. They are associated with beneficial effects on lowering the risk of fractures amongst a broad age range of patients; even those living with frailty [92–94]. Evidence shows 10 mg of alendronate daily for 10 years increased bone mineral density by 13.7% at the lumbar spine, 10.3% at the trochanter, 5.4% at the femoral neck, and 6.7% at the total proximal femur. Importantly, both oral and intravenous bisphosphonate therapy have shown to reduce the risk of mortality when commenced as secondary prevention measures after a fracture [93–96].

UK NICE guidance [97] recommends Alendronate 10 mg once daily or 70 mg once weekly; or Risedronate 5 mg once daily or 35 mg once weekly, for postmenopausal women and men over 50 years of age, who have confirmed osteoporosis on DXA. Evaluation of BMD usually occurs between 3 and 5 years. Thereafter, treatment is continued if the patient continues to be risk of fracture or has commenced on corticosteroid therapy. If the T-score is > − 2.5, a drug holiday may be recommended pending further evaluation of BMD and fracture risk. However, discontinuation of bisphosphonates in postmenopausal women at this time may be associated with up to 40% higher risk of new clinical fractures compared to those who continue bisphosphonates [98]. Ibandronic acid is not recommended first-line.

Adverse effects of oral bisphosphonates include gastrointestinal symptoms, bone/joint pain, oesophageal ulceration, and rarely osteonecrosis of the jaw (the highest risk is in patients with cancer). Atypical femoral fractures can occur particularly after 5 years of bisphosphonate use at the rate of 1:1000/year. Oral bisphosphonates should be taken on an empty stomach, in an upright position, with a glass of water [99]. Adherence to bisphosphonates may be challenging in older people because of this complex dosing regime and can be compounded by the presence of polypharmacy, impaired cognition and physical care needs. Furthermore, bisphosphonates are not stable to be kept in compliance aids. In older people with severe gastro-oesophageal reflux, dysphagia or cognitive impairment, alternative preparations, i.e., intravenous (IV) yearly Zoledronic acid or alternatives to bisphosphonates may be used [66]. Bisphosphonates are renally excreted and should be avoided in renal impairment. Estimated glomerular filtration rates (eGFR) provide thresholds to base treatment decisions upon. For example, alendronate and risedronate should be avoided when creatinine clearance is below 35 mL/min/1.73 m^2^ and 30 mL/min/1.73 m^2^, respectively. However, eGFR may not be accurate in older people, especially those living with frailty and sarcopenia. Cockcroft and Gault estimation of GFR is, therefore, appropriate to use in these situations; especially when IV Zoledronic is being considered (Table 2).

##### Denosumab

Denosumab is a humanized monoclonal antibody that blocks RANKL and hence osteoclastic activity. It is given via a subcutaneous injection (60 mg)on a 6-monthly basis alongside calcium and vitamin D supplementation in individuals with a GFR > 30 ml/min/1.73 m^2^. FREEDOM (Fracture Reduction Evaluation of Denosumab), a large multicentre placebo-control trial showed a reduction in fracture incidence of 68% for vertebral fractures, 40% for hip fractures, and 20% for non-vertebral fractures, in the first 3 years, in postmenopausal woman taking Denosumab [100]; 10 year follow up showed continued decreasing fracture incidence and an increase in BMD without plateau [101]. Denosumab is often used as an alternative when oral bisphosphonates are not tolerated, are contraindicated or other social and psychological problems preclude bisphosphonate therapy. Treatment is usually for 5–10 years. The anti-resorptive effects of Denosumab rapidly diminishes after treatment cessation and consequently increase fracture risk back to pre-treatment levels within 12 months of cessation and therefore, requires both patient and physician led reminders on a 6-monthly basis. This is in contrast to bisphosphonates where BMD is maintained for at least 2 years after treatment withdrawal. Side effects include hypocalcaemia especially in individuals with impaired renal function, skin rash, increased risk of bacterial infections, osteonecrosis of the jaw and rarely, atypical femoral fractures (Table 2).

When initiating Denosumab or other anti-resorptive therapy, it is important to ensure that patients have normal serum calcium levels and are replete in vitamin D [66]. This lowers the risk of severe hypocalcaemia during treatment. Multiple loading regimes exist for those who are Vitamin D deficient. In the authors’ clinical practice, 100,000 IU of colecalciferol for individuals living with frailty and where rapid loading is needed appears to be well tolerated. Alternatives include 20,000 IU three times a week followed by 800 IU—1000 IU/day to maintain a serum vitamin D level above 50 nmol/L. Vitamin D in excess is associated with hypercalcemia, hypercalciuria and mineral deposits in soft tissues. However, doses of 800 IU to 1000 IU /day the for the prevention of Vitamin D deficiency is considered safe [102].

#### 3. Hormonal treatment

##### Selective oestrogen receptor modulators (Raloxifene and Lasoxifene)

Selective oestrogen receptor modulators (SERMs) such as Raloxifene and Lasoxifene aim to prevent bone resorption due to oestrogen deficiency. They are indicated primarily for the treatment and prevention of osteoporosis in post-menopausal women and are indicated after first line therapies have been considered. As an example of efficacy, Lasoxifene 0.5 mg showed 42% reduction in vertebral fracture risk and 24% reduction in hazard rates of non-vertebral fractures, at 3 years in women aged 59–80 years [86, 87]. Most common reported side effects include hot flushes and lower limb cramps. Increased risk of venous thromboembolism is the most severe adverse effect, though fortunately rare (Table 2).

##### Testosterone

Endocrine Society recommends testosterone for men at high risk for fracture with testosterone levels below 200 ng/dl (6.9 nmol/l). This should be considered even for patients who lack standard indications for testosterone therapy but who have contraindications to other osteoporosis’ therapies [103]. Potential side effects include cardiovascular and metabolic effects and rises in prostate specific antigen [104] (Table 2).

##### PTH analogues (Teriparatide, Abaloparatide)

Teriparatide, a synthetic parathyroid hormone, is anabolic in bone rather than anti-resorptive. It can be used in men and women who are intolerant or who suffer severe side effects from first line therapies described. Teriparatide should be administered subcutaneously, 20mcg daily for a maximum of 24 months. Teriparatide is contraindicated in patients with metabolic bone diseases such as Paget’s disease, skeletal muscle metastases or previous bone radiation therapy. Side effects include nausea, pain in limbs, headache and dizziness. Abaloparatide, a newer PTH analogue showed lower risks of new vertebral fractures when compared to both placebo and teriparatide as well as lower risk of nonvertebral fractures in comparison to placebo and a significant increase in BMD amongst 2463 post-menopausal women aged 49–86 years in the The ACTIVE study [105] (Table 2).

#### 4. Novel therapies

##### Romosozumab

Romosozumab is a monoclonal antibody that binds sclerostin leading to increased bone formation and a decrease in bone resorption. It is administered as a monthly subcutaneous injection, at a dose of 210 mg. The FRAME study was an international, randomized, double-blind, placebo-controlled trial that compared Romosozumab with placebo in postmenopausal women aged 55–90 with osteoporosis. Both groups also received denosumab 6 monthly. The Romosozumab treatment arm showed a 75% lower risk of new vertebral fractures, at 24 months; with no significant difference in adverse events [106].

The ARCH study, however, compared a group of postmenopausal women that received alendronate for 24 months and a group that received Romosozumab for 12 months followed by alendronate for another 12 months. Interestingly, patients on the Romosozumab-to-alendronate group had a 48% lower risk of new vertebral fractures (*p* < 0.001) and 27% lower risk of clinical fractures (*p* < 0.001). The risk of nonvertebral fractures was lower by 19% (*p* = 0.04) and the risk of hip fracture was lower by 38% (*p* = 0.02). Nonetheless, it is important to note an imbalance in serious cardiovascular adverse events between the 2 groups—16 patients (0.8%) in the Romosozumab group vs 6 (0.3%) in the alendronate group reported cardiac ischemic events (odds ratio 2.65; 95% CI 1.03–6.77); and 16 patients (0.8%) in the Romosozumab group vs 7 (0.3%) in the alendronate group reported cerebrovascular events (odds ratio 2.27; 95% CI 0.93–5.22). Further studies are needed to clarify this imbalance [107] (Table 2).

##### Dual inhibition of Dickkopf-1 (Dkk1) and sclerotin

Dkk1 is one of the antagonists in the Wnt signalling pathway which is an important cascade involved in bone formation. It was found that inhibition of sclerotin can lead to an upregulation of Dkk1 expression. Based on this, a study demonstrated the use of an engineered bio-specific antibody against sclerostin and Dkk1 simultaneously resulted in a bigger effect on bone formation compared to monotherapies in both rodents and primates. Improvements in healing and repair capacity of fractured bones were also seen when dual inhibition was used [108]. Results from clinical trials are currently awaited.

### Treatments for osteosarcopenia

There are biochemical and hormonal relationships between bone and muscle through molecular cross-talk between myokines, osteokines and adipokines, secreted from muscle cells, bone cells and marrow adipose tissue, respectively. Abnormal expansion of marrow adipose tissue has been postulated to be a significant factor in the progression of post-menopausal osteoporosis [109, 110]. Similarly, myosteatosis negatively impacts on muscle quality, the force generated per skeletal muscle unit area. Although no single molecule has been implicated in the pathogenesis of either condition, ongoing research may provide new targets for future therapy. Growth hormone, insulin-like growth factor-1, gonadal sex hormones, vitamin D and myostatin have all been associated with a delay in the onset of osteosarcopenia [111]. Though postulated to be novel therapeutic targets for drug development, currently no pharmacological treatments exist for osteosarcopenia [112].

### Dementia and fragility fracture

Dementia increases with older age and is characterised by the presence of multimorbidity, cognitive and behavioural problems, visual and motor problems that consequently increase the risk of falls. Furthermore, high prevalence of malnutrition, frailty and sarcopenia in patients with dementia increases the likelihood of osteoporosis. This coexistence poses a particular therapeutic challenge, and an identified need exists for bone health measurement and pharmacological management in patients with dementia who are at high risk of incident and future fracture [113]. However, patents living with dementia are least likely to have their fracture risk assessed or receive longer term secondary prevention medications due to such reasons as delirium, worsening cognitive decline, institutionalisation, poor adherence and competing polypharmacy. In addition, altered pharmacokinetics conspire to risk adverse drug reactions (ADR) in this group of patients. In this regard, fracture risk undoubtedly increases commensurate with the incidence of dementia. CGA for such patients may just identify achievable goals to attain in the short and medium term when risks and benefits of treatment are considered in context of the wider social, physical and psychological domains [69].

## Conclusions

The incidence of osteoporosis increases with age and the prevalence is increasing in line with global population ageing. Osteoporosis and sarcopenia often coexist and are associated with substantial burden for older people in terms of morbidity and mortality. Both are often underdiagnosed and undertreated. Routine assessment of bone and muscle health should be part of a holistic multidisciplinary led, personalised comprehensive geriatric assessment both in primary and secondary care. Nutrition, physical activity, exercise, gait and balance interventions have been shown to be beneficial for bone and muscle health and in reducing the number of falls. These should be instituted alongside other lifestyle measures as part of the treatment strategy for an older person.

Older people at risk of fracture derive considerable benefits from treatment with bone sparing agents; the choice should take into account frequency, route of administration, cost, potential for polypharmacy, ADR and long-term survival. In clinical practice bisphosphonates and denosumab; either first line or for older people intolerant to bisphosphonates, have a strong evidence base for efficacy in older people. For those intolerant or who are unable to have bone sparing agents, calcium and vitamin D should be offered to maintain bone health.

## Supplementary Information

Below is the link to the electronic supplementary material.

Supplementary file1 (DOCX 278 KB)
