# Supplementary material for: Pathophysiology and treatment of osteoporosis: challenges for clinical practice in older people
Source: Aging Clin Exp Res. 2021 Mar 20;33(4):759–73. doi: 10.1007/s40520-021-01817-y (PMC8084810; doi:10.1007/s40520-021-01817-y)
Supplement: Supplementary file 1 — Supplementary file1 (DOCX 278 KB) [file 40520_2021_1817_MOESM1_ESM.docx]

**Supplementary information 1**

**Methods**

We performed a literature review using medical journal databases, including PubMed and the Cochrane Library. Search terms pertinent to this topic used included “bone development”, “osteoporosis”, “treatment”, “pathophysiology”, “osteosarcopenia” “older people”. Article titles and abstracts were then assessed for relevance and full-text screening performed if the title or abstract contained one or more search terms and the article itself was deemed relevant to this topic. Articles were also selected for review based on the authors own clinical expertise and knowledge of pre-existing work in this field. Only English language published articles were included. We applied the principles from the Scale for the Assessment of Narrative Review Articles (SANRA) to this manuscript (5) (supplementary table 1). The score was 10/12 indicating a higher quality narrative review.

| **Scale for the Assessment of Narrative Review Articles - SANRA** | | | |
| --- | --- | --- | --- |
| **1** | **Justification of the article’s importance for the readership** | | |
|  | The importance is not justified | 0 |  |
|  | The importance is alluded to, but not explicitly justified | 1 |  |
|  | The importance in explicitly justified | 2 | X |
| **2** | **Statement of concrete aims or formulation of questions** | | |
|  | No aims of questions are formulated | 0 |  |
|  | Aims are formulated generally but not concretely or in terms of clear questions | 1 |  |
|  | One or more concrete aims or questions are formulated | 2 | X |
| **3** | **Description of literature search** | | |
|  | The search strategy is not presented | 0 |  |
|  | The literature search is described briefly | 1 | X |
|  | The literature search is described in detail, including search terms and inclusion criteria | 2 |  |
| **4** | **Referencing** | | |
|  | Key statements are not supported by references | 0 |  |
|  | The referencing of key statements in inconsistent | 1 |  |
|  | Key statements are supported by references | 2 | X |
| **5** | **Scientific reasoning (e.g. incorporation of appropriate evidence, such as RCTs in clinical medicine** | | |
|  | The article’s point in not based on appropriate arguments | 0 |  |
|  | Appropriate evidence in introduced selectively | 1 |  |
|  | Appropriate evidence is generally present | 2 | X |
| **6** | **Appropriate presentation of data (e.g. absolute vs relative risk; effect sizes without confidence intervals)** | | |
|  | Data are presented inadequately | 0 |  |
|  | Data are often presented in the most appropriate way | 1 | X |
|  | Relevant outcome data are generally presented appropriately | 2 |  |
|  |  |  |  |
|  | **Sum score** | **10** |  |

**Supplementary table 1**

**
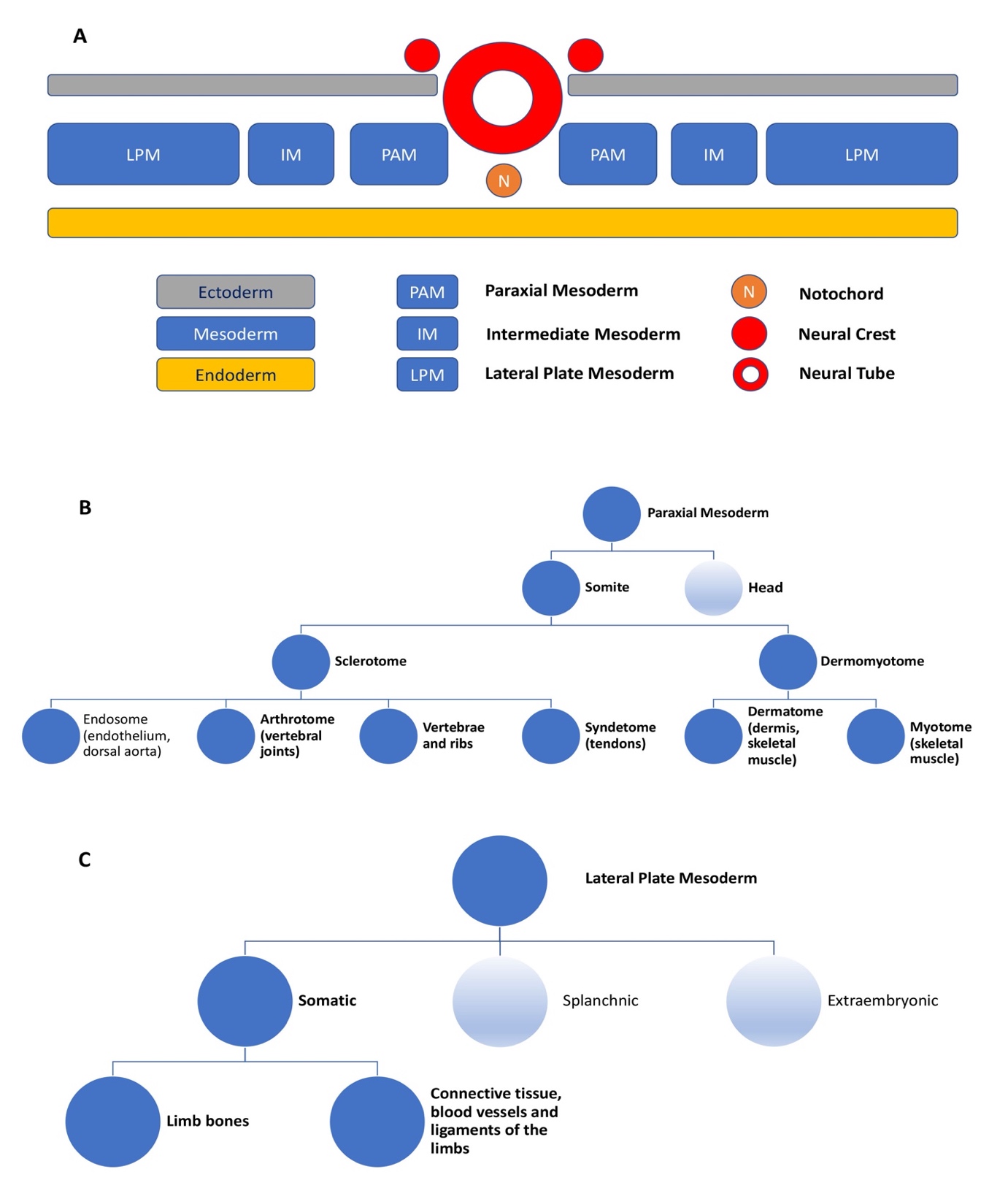
**

**Supplementary figure 1. The development of bone A.** Transverse section schematic demonstrating the trilaminar embryo formed during gastrulation, with the major divisions of the embryonic mesoderm. **B & C**. Lineages of the embryonic mesoderm that demonstrate the common origins of bone and muscle
